# Machine Learning Regression-Enhanced Property Prediction of Brush Particle Solids

**DOI:** 10.1021/acsapm.6c03454

**Published:** 2026-10-13

**Authors:** Kevin Ferguson, Ayesha Abdullah, Yuqi Zhao, Yunhao Xu, Michael R. Bockstaller, Levent Burak Kara, Lawrence Drummy, Eric Harper, Andrew Gillman

**Affiliations:** † Department of Mechanical Engineering, Carnegie Mellon University, 5000 Forbes Ave, Pittsburgh, Pennsylvania 15213, United States; ‡ Department of Materials Science and Engineering, Carnegie Mellon University, 5000 Forbes Ave, Pittsburgh, Pennsylvania 15213, United States; § Materials & Manufacturing Directorate, Air Force Research Laboratory, 2977 Hobson Way, Wright-Patterson AFB, Ohio 45433, United States

**Keywords:** polymer brush, nanoparticle, modulus, machine learning, regression, property prediction

## Abstract

The development of advanced hybrid materials such as polymer-grafted nanoparticles (aka “brush” or “hairy” particles) hinges on the ability to accurately predict suitable compositions to realize desired functionalities. Such systems are characterized by expensive synthesis, sparse data, and large parameter spaces that often render physics-based modeling or experimental exploration of structure–property relationships expensive and impractical. Regression models provide a versatile data-driven alternative to establish empirical structure–property relations. However, the implications of sparse data availability and method of training on the predictive capability of regression models outside of the respective range of training data is poorly understood. This work presents a comparative analysis of widely used linear and nonlinear models versus machine learning regression models to evaluate their ability to predict the elastic modulus of brush particle solids formed by poly­(methyl methacrylate) (PMMA) and polystyrene (PS) tethered silica nanoparticles with systematic variation of the degree of polymerization, dispersity, and grafting density. The methods investigated include k-nearest neighbors, multilayer perceptron, and XGBoost regression. The performance of each model is assessed on a consistent set of training and testing data. Within the experimental certainty, the k-nearest neighbors regressor and XGBoost were found to provide the most reliable property prediction. Combining data sets of disparate polymer systems (PMMA/PS) was found to increase the accuracy of prediction and to enable models to forecast previously reported unexpected behavior outside of the range of the original training data. This supports the use of trained regression models as capable exploratory tools to reveal structure–property relations for systems that are controlled by multiple parameters with unknown interactions.

## Graphical abstract

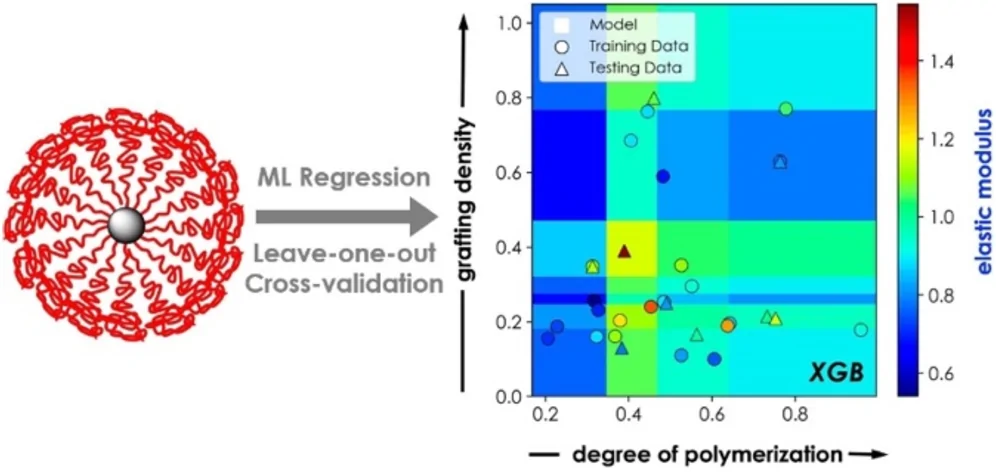

## Introduction

Advanced materials for use in applications such as energy storage and generation, electronics, or biomedical engineering are often composite materials featuring different components that interact to enable desired functionalities.^1–5^ One example is brush particle solids, i.e., materials assembled from polymer-tethered particles or colloids (aka “brush” or “hairy” particles) that have emerged as a platform for hybrid materials with novel property combinations such as optical transparency, high dielectric constant and breakdown strength, or novel photonic and phononic characteristics.^6–8^ The emergence of novel properties in this class of materials has been related to the uniform microstructure and the impact of steric confinement (that results from the crowding of chains at the interface) on the conformation of grafted chains and its impact on the assembly behavior and interactions between brush particles in the solid state. Experimental and simulation studies have shown physical properties of brush particle solids to depend on the chain degree of polymerization (*N*), grafting density (σ), dispersity (*Đ*), particle diameter (*d*) as well as composition. However, while trends of the impact of select parameter variation on the physical properties can generally be rationalized, the interaction between the various parameters and the cumulative effect of multiple parameter variation on the absolute property values remain unknown.^9–16^ This renders the deliberate design of brush particle materials with target properties difficult if properties are to be optimized with respect to more than one parameter. One example might be the fabrication of brush particle hybrids with a balance of elastic modulus *and* inorganic content (which is a desirable goal in applications such as dielectric separators, luminescent films, or high refractive index glasses).^6,8^ Designing an “optimal” brush particle hybrid material is then a multiobjective optimization problem. Given that methods for brush particle synthesis such as surface-initiated reversible–deactivation radical polymerization (as well as quantitative estimation of properties through simulation) are expensive, iterative variation of parameters is impractical; this renders the prediction of suitable brush particle compositions that match desired properties a challenge.

Optimization problems in high-dimensional spaces are excellent candidates for machine learning (ML) enabled explorations into material design. Application of ML was traditionally focused on fields with “big data” (e.g., image feature recognition), but the permeation of ML into materials property prediction has drawn attention to problems that do not generally satisfy big data requirements.^17^ To observe trends based on available testing data, methods such as linear or polynomial regression are commonly applied; however, these methods are established for low-dimensional regression problems and observe linear or polynomial relations between variables. In contrast, ML methods such as *k*-nearest neighbors (KNN) act as nonparametric estimators that predict values based on neighboring data points, without making explicit assumptions about underlying relationships within the data.^18^ In the case of advanced engineering materials such as brush particle solids (or similar hybrid materials), in which properties frequently depend on a range of parameters with unknown functional relationships, this holds the promise of more accurate property prediction. For example, in the case of brush materials, prior research has shown ML regression models to afford insights into structure–surface energy relationships and physicochemical properties of polymer-grafted surfaces.^19–23^

To realize the potential benefits provided by ML-enabled classification and regression methods, research needs to better understand the implications of the limited volume and distribution of data—which is typical for experimental systems—on the predictive capability and practicality of ML models. In this work, ML regression methods were used to predict the elastic modulus of a library of brush particle solids composed of poly­(methyl methacrylate) (PMMA) and polystyrene (PS) grafted silica nanoparticles (particle diameter *d* = 7.9 *±* 2.2 nm). A library of 76 (42 PMMA- and 34 PS-) brush particle systems with systematic variation of structural parameters (*N*, σ, *Đ*) was synthesized using surface-initiated activators regenerated by electron transfer atom transfer radical polymerization (SI-ARGET ATRP) following previously published methods and the modulus of annealed thick (∼0.5 mm) films was determined after annealing (120 °C, 8 h).^24^ Several well-studied ML regression techniques were compared in their ability to predict the Young’s modulus (*E*). Basic regression methods, including ordinary least-squares (OLS) and polynomial regression (PR), served as benchmarks to evaluate the performance of ML algorithms. Selected ML methods included *k*-nearest neighbors (KNN), support vector regression (SVR), decision tree (DT), random forest (RF), extreme gradient boosting (XGBoost), and a multilayer perceptron (MLP). These methods were previously reported to successfully predict properties of materials based on limited experimental data sets and thus hold promise in this area of application.^19–23,25–28^ Examination of these sparse data sets with ML can enable novel insights into structure–property relations and thus inform future experimental endeavors.^29–31^

## Materials and Methods

In this section, the data set and ML models are presented in detail. Descriptions of the material composition, initiator surface modification, brush synthesis procedure, and mechanical testing methods are included.

### Materials

Monomers (styrene, S (99%), Aldrich; methyl methacrylate, MMA (99%), Aldrich) were purified by passing through a column filled with basic alumina to remove inhibitor. Other chemicals, tris­(2-dimethylaminoethyl)­amine (Me6TREN, 99%, Alfa), 4,4’-dinonyl-2,2’-bipyridine (dNbpy, 97%, Aldrich), anisole (99%, Aldrich), tetrahydrofuran (THF, 99%, VWR), methanol (99%, VWR), hexane (99%, VWR), acetone (99%, VWR), *N*,*N*-dimethylformamide (DMF, 99%, VWR), 2-bromoisobutyryl bromide (2BiB, Aldrich, 98%), triethylamine (TEA, Aldrich, 99.5%), copper­(II) bromide (CuBr_2_, 99%, Aldrich), copper­(II) chloride (CuCl_2_, 99%, Aldrich), copper­(I) chloride (CuCl, 97%, Sigma-Aldrich), tin­(II) 2-ethylhexanoate (Sn­(EH)_2_, 95%, Aldrich), hexane (Fluka), 48% hydrofluoric acid aqueous solution (HF, >99.99%, Aldrich), ammonium hydroxide aqueous solution (NH_4_OH, 28.0–30.0%, Fisher), anhydrous magnesium sulfate (MgSO_4_, Fisher), and hexamethyldisilazane (HMDZ, Aldrich, 99%) were used as received. Silica NPs (SiO_2_) 30 wt % dispersion in methyl isobutyl ketone (MIBK-ST) were donated by Nissan Chemical America Corp. The particle diameter (*d* = 7.9 *±* 2.2 nm) was measured by transmission electron microscopy (TEM). The surface initiator densities were moderated with a dummy initiator chlorotrimethylsilane (99%, Aldrich) as described in previous publications.

### Tetherable Initiator

3-(Chlorodimethylsilyl)-propyl 2-bromoisobutyrate (BiBSiCl) was used as a tetherable initiator. The synthesis of the material as well as the coupling of initiator to the particle surface was described previously.^24^ Appropriate stoichiometric blending with nonreactive trichlorosilane was used to control the grafting density.

### Particle Brush Synthesis

Activators Regenerated by Electron Transfer (ARGET) SI-ATRP was used because of its more resilient features, especially at reduced catalyst concentration, and slower kinetics. Initiator modified silica colloids (SiO_2_–Br), monomers (styrene, MMA), solvents (anisole, DMF), CuBr_2_, and Me6TREN were mixed thoroughly in a sealed Schlenk flask. Meanwhile, a stock solution of Sn­(EH)_2_ in anisole was prepared. Both mixtures were degassed by nitrogen purging; then, the Sn­(EH)_2_ solution was injected into the Schlenk flask to activate the catalyst complex, and the flask was immediately put into an oil bath. For a detailed procedure, including the characterization of polymer brush parameters (σ, *N*, Đ), the referenced work by Pyun and Matyjaszewski can be consulted.^32^ After suitable time of polymerization (i.e., conversion ratios), according to the volumes of solutions (corresponding to about 100 mg of material each) were sequentially extracted, purified, and molecular characteristics were determined. This enabled the preparation of a library of materials with systematically varied brush characteristics. Compositional parameters of all materials are provided in Tables 1 and 2. For all systems, the dispersity of tethered chains was in the range of 1.09 *≤ Đ ≤* 2.04.

**Table 1:** Molecular Characteristics of SiO_2_–PMMA Systems^a^

index	*N*	σ, chains/nm^2^	*Đ*	*E*, normalized
1	482	0.589	1.28	0.6784
(2)	763	0.629	1.32	0.8165
3	763	0.629	1.32	0.7724
(4)	313	0.348	1.25	1.1271
5	313	0.348	1.25	1.0993
6	526	0.351	1.24	0.9775
7	526	0.351	1.24	1.1075
8	367	0.161	1.25	1.1050
9	642	0.196	1.30	0.9456
10	642	0.196	1.30	0.8615
11	957	0.178	1.30	0.9236
12	957	0.178	1.30	0.8075
13	484	0.253	2.04	0.7389
14	484	0.253	2.04	0.8860
15	484	0.253	2.04	0.7593
16	551	0.295	1.59	0.9334
17	551	0.295	1.59	0.9440
18	228	0.187	1.68	0.7348
19	228	0.187	1.68	0.8484
20	317	0.256	1.76	0.5403
21	317	0.256	1.76	0.5860
22	327	0.231	1.63	0.6988
23	327	0.231	1.63	0.7094
24	605	0.100	1.99	1.0895
25	605	0.100	1.99	0.7487
(26)	383	0.130	1.61	0.7871
27	526	0.110	1.19	1.0151
28	526	0.110	1.19	0.8190
29	453	0.240	1.68	1.3543
30	323	0.160	1.90	0.8835
(31)	490	0.250	1.38	0.8124
(32)	389	0.390	1.60	1.5439
33*	206	0.155	1.35	0.7104
34*	379	0.203	1.22	1.2131
35*	405	0.685	1.18	0.8856
36*	445	0.763	1.16	0.8793
(37)*	460	0.799	1.18	1.0665
(38)*	563	0.166	1.13	0.9897
39*	637	0.189	1.25	1.2817
(40)*	732	0.215	1.13	1.0013
(41)*	752	0.209	1.24	1.1581
42*	777	0.770	1.15	1.0485

a Definition of parameters: Degree of polymerization (*N*), grafting density (σ), Young’s modulus (*E*) normalized with respect to linear PMMA (*M*_n_ = 300 000 g/mol), dispersity (*Đ*). Indices within parentheses are points that were randomly chosen as test points, rather than training points. Points whose indices contain asterisks (*) had modulus measured via nanoindentation rather than tensile testing.

**Table 2:** Molecular Characteristics of SiO_2_–PS Systems^a^

index	*N*	σ,/nm^2^	*Đ*	*E*, normalized
1	120	0.784	1.17	0.5589
2	136	0.845	1.11	0.6214
3	188	0.705	1.21	0.9456
4	204	0.740	1.10	0.5158
5	212	0.723	1.14	1.2811
6	213	0.694	1.18	0.9309
7	226	0.741	1.11	1.0862
8	231	0.620	1.20	0.9540
9	231	0.781	1.14	1.2499
10	355	0.613	1.10	1.2267
11	365	0.706	1.15	1.2888
12	365	0.584	1.22	1.1077
13	382	0.789	1.47	0.8994
14	400	0.606	1.25	1.0060
15	432	0.745	1.36	0.9806
16	549	0.455	1.35	1.1592
17	638	0.499	1.72	1.2528
18	762	0.486	1.18	1.0168
19	829	0.602	1.25	1.0841
20	841	0.475	1.28	1.1272
21	890	0.449	1.25	0.8447
22	1087	0.334	1.28	1.2782
23	1777	0.189	1.19	0.9561
24	1800	0.218	1.42	1.0657
25	221	0.172	1.09	0.4430
26	255	0.118	1.11	0.5029
27	328	0.159	1.14	0.6913
28	440	0.123	1.15	1.0255
29	758	0.102	1.10	1.1235
30	1103	0.074	1.24	1.1090
31	432	0.033	1.21	1.6616
32	600	0.038	1.29	1.5021
33	954	0.027	1.33	1.4059
34	1576	0.025	1.47	1.2454

a Definition of parameters: Degree of polymerization (*N*), grafting density (σ), Young’s modulus (*E*) normalized with respect to linear PS (*M*_n_ = 300 000 g/mol), dispersity (*Đ*).

### Mechanical Analysis

Tensile testing of bulk brush particle films was performed using a DMA (TA RSA-G2) operating in tensile mode. The film thickness was between 100 and 200 μm. Films were cast from 3% toluene solution and annealed at *T* = 130 °C for 24 h prior to testing. In tensile tests, samples were stretched at a constant tensile rate of 0.05 s*–*1 at 23 °C. Nanoindentation tests were performed on >20 μm thick films that were prepared from concentrated solutions (∼20 mg/mL) using PTFE molds. After annealing (*T* = 130 °C, 24 h), the elastic modulus of films was determined using an MTS nanoindenter XP with a Berkovich indenter. Displacement was less than 10% of the particulate film thickness to exclude any effect from substrates. At least nine locations were indented with 500 nm depth limit, 5 nm/s displacement rate, 0.05 nm/s allowable drift rate, and 10 s constant loading time before unloading for each sample. Distinct locations were tested to avoid possible artifacts due to film inhomogeneity. The standard deviation of the measurements was calculated as the experimental error. Values of Young’s modulus that were determined from nanoindentation (Table 1) represent average values across the distinct indentation locations.

### Training of Regression Models

The grafting density σ and the degree of polymerization *N* were used as input parameters of the ML regression models while the corresponding Young’s modulus *E* served as the predicted property. Nine of the data points presented for PMMA were randomly selected as testing data—the remainder of the PMMA data was for training (see Figure 1a). The model-training process involved performing hyperparameter optimization with leave-one-out (LOO) cross-validation on the PMMA data set to minimize validation mean absolute percentage error (MAPE). The latter was chosen as metric to gauge training progress due to the reduced sensitivity of MAPE to outliers. In several cases, the root-mean-square error (RMSE) was used to confirm consistent results independent of the metric used for training (for a definition of MAPE and RMSE, please see the Supporting Information). Results were compared with training results using nested cross-validation to ascertain the influence of cross-validation process. All calculations were carried out using Python’s Scikit-Learn implementation, with the exception of XGBoost, which used its official Python package.^33,34^ Six distinct regression methods, including *k*-nearest neighbors (KNN); tree-based models decision tree (DT), random forest (RF), and XGBoost (XGB); and gradient-descent based methods Support Vector Regression (SVR) and Multilayer Perceptron (MLP), were evaluated. Ordinary least-squares (OLS) and polynomial regression (PR) were applied as benchmark techniques to observe the performance of other models against standard regression techniques. Overfitting was evaluated using the difference between training and validation/test errors for each model and each split (i.e., the generalization gap). A large positive Δ­(RMSE)/Δ­(MAPE) (with Δ­(*X*) = *X*_test_ – *X*_train_, with *X* = RMSE, MAPE) was interpreted as indicative of overfitting. In contrast, small and stable gaps across folds indicated that the model generalized well.

**Figure 1:**
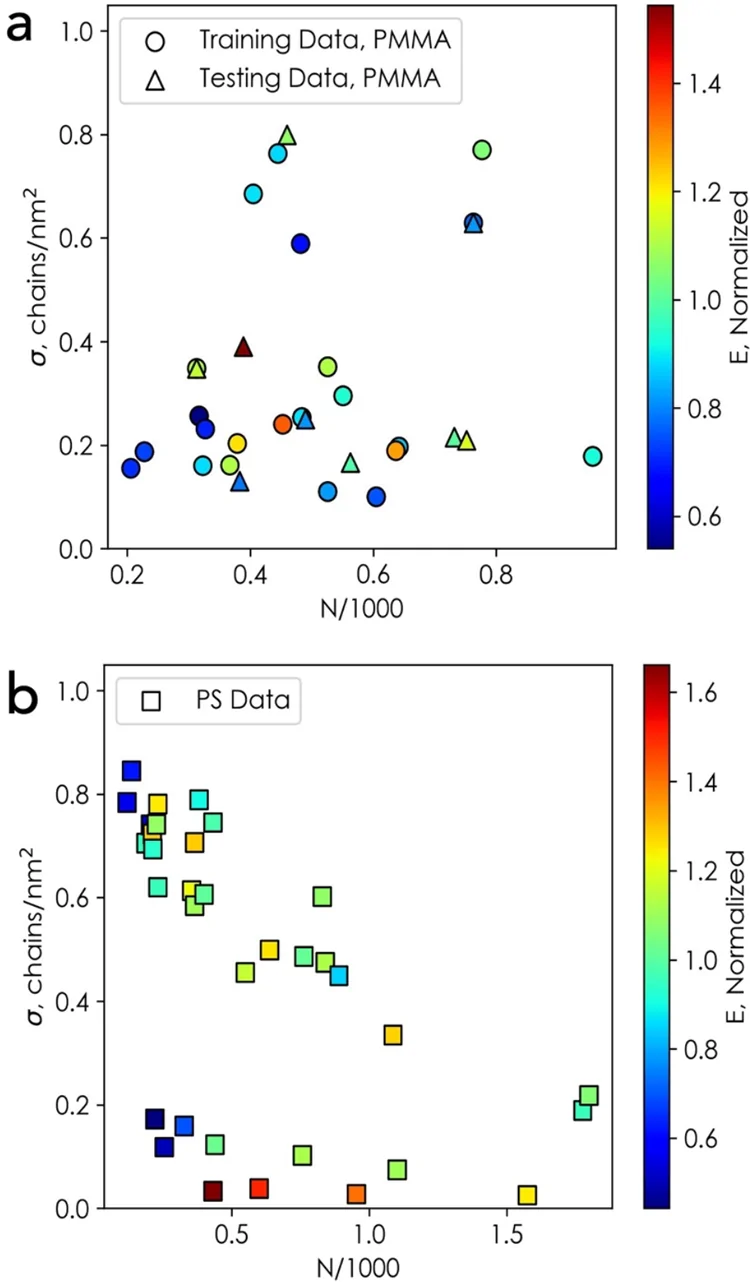
Data for elastic (Young’s) modulus *E* of SiO_2_PMMA (a) and SiO_2_PS (b) brush particle systems. Values are normalized with respect to respective values of linear reference PMMA (a) and PS (b) polymer. Modulus values are shown as a function of structural parameters σ and *N*.

## Results and Discussion

A total of 42 SiO_2_–PMMA and 34 SiO_2_–PS systems were synthesized using SI-ARGET ATRP. For all systems, the dispersity of tethered chains was in the range of 1.09 *≤ Đ ≤* 2.04. Compositional parameters of all materials are provided in Tables 1 and 2.

The elastic modulus *E* of materials was determined by two methods. First, from elastic recovery using nanoindentation as described in previous work.^35,36^ Second, for select PMMA-grafted brush particle solids (free-standing films, thickness *>* 200 μm), *E* was also determined through tensile testing for confirmation (samples tested by nanoindentation are identified by “*” symbols in Table 1). Tensile stress–strain measurements were considered as the standard method for modulus determination; however, due to the large amount of material needed, only a subset of materials was evaluated to confirm nanoindentation results. For the samples tested, the two methods were found to yield congruent results both in terms of trend and magnitude of *E* after normalization with the modulus measured for linear PMMA and PS (*M*_n_ = 300,000) reference polymers using the same methodology. The results are summarized in normalized form in Tables 1 and 2.

Figure 1 depicts the full data sets of the normalized elastic modulus for SiO_2_–PMMA (Figure 1a) and SiO_2_–PS (Figure 1b) material systems as a heatmap over varied ranges of σ and *N*. Note that the influence of dispersity on *E* was found to be negligible. This was consistent with prior reports that demonstrated the elastic modulus of brush particle solids to be determined by dispersion interactions between adjacent brush particles.^37^ Since the latter depend primarily on the volume fraction of repeats and brush interdigitation, the impact of chain dispersity on the elastic modulus was not expected to be significant (note that this does not apply to the plastic regime). Therefore, at constant particle core size and chain chemistry, the primary components influencing *E* of brush particle solids was expected to be σ and *N*. Dispersity was thus excluded as a parameter in the following discussion.

Each regression method, as discussed in the previous section, had its hyperparameters optimized using LOO cross-validation. *k*-nearest neighbors (KNN) is a nonparametric regression technique that, rather than learning a set of parameters, makes predictions on new points by averaging the output values of the nearest *k* points in the training set. During hyperparameter optimization, the value of *k* and whether to apply distance-based weights while averaging, were optimized. Three tree-based methods of increasing complexity were explored: decision trees, random forests, and XGBoost. These are efficient learners for classification and regression problems. The simplest tree model is the decision tree (DT), which identifies a splitting attribute based on the reduction of the squared error and can continue to split the data until a maximum depth value (this maximum depth was treated as a hyperparameter). A more complex variant of the decision tree, the random forest (RF), was also trained. RF models are ensembles of trees, each using only a subset of the data and its features. The final prediction is an average of all individual tree estimator predictions. Maximum tree depth and number of estimators were the targets for hyperparameter optimization for the RF model. The last tree-based method, XGBoost (XGB), is a “boosting” method designed to train subsequent models based on prior residuals, resulting in a sum of those models; this method is notable for its ability to handle complex relationships in data.^36,38^ Learning rate, maximum tree depth, and number of tree estimators were optimized as hyperparameters. Two distinct ML methods stand out for higher-dimensional regression problems, including support vector regression (SVR) and multilayer perceptrons (MLP). Support vector machines are traditionally used to split data sets into distinct classes. However, applying kernels can represent nonlinear boundaries and extends application to regression problems; regularization strength was optimized as a hyperparameter, and a radial basis function kernel was applied. Finally, MLPs are widely lauded for their value as universal function approximators (i.e., any input–output relationship can theoretically be represented with a sufficiently large MLP).^39^ For the validation study, the MLP was optimized with respect to the size and number of hidden layers and the activation function applied to those layers (“tan h” or “ReLU”). The results of hyperparameter optimization using LOO cross-validation for each of the regression models are summarized in the Supporting Information, Table S1. Training and testing results are summarized in Table 3. The fundamental metric used to evaluate model performance was mean absolute percentage error (MAPE); the root-mean-squared error (RMSE) is presented as an additional metric for completeness. Both parameters are defined in the Supporting Information. Table 3 also includes the results for evaluating the trained model on PS data; this will be discussed separately.

**Table 3:** MAPE and RMSE of Each Model on Training (PMMA), Testing (PMMA), and PS Data (Using LOO Cross-Validation)

	train (PMMA)		test (PMMA)		test (PS)	
model^a^	MAPE	RMSE	MAPE	RMSE	MAPE	RMSE
DT	15.21%	0.169	18.16%	0.261	**23.03%**	0.284
KNN	**4.34%**	**0.059**	**12.36%**	0.243	25.72%	0.292
MLP	13.26%	0.151	17.45%	0.278	29.91%	0.332
OLS	17.08%	0.185	17.11%	0.270	25.81%	0.296
PR	17.19%	0.187	17.18%	0.263	26.44%	0.302
RF	12.90%	0.140	14.73%	0.225	23.26%	**0.269**
SVR	16.43%	0.188	17.61%	0.281	28.30%	0.325
XGB	15.89%	0.173	17.78%	0.262	26.15%	0.306

a Abbreviations: DT, decision tree; KNN, *k*-nearest neighbor; MLP, multilayer perceptron; OLS, ordinary least-squares; PR, polynomial regression; RF, random forest; SVR, support vector regression; and XGB, extreme gradient boosting.

Table 3 reveals that none of the models could predict *E* for unseen architectures with outstanding accuracy on both the PMMA training and testing data. All models produced MAPE values on testing data below 20%, with DT performing the worst (18.1%) and KNN performing the best (12.4%). The tree-based ensembles, RF and XGB, performed similarly, with MAPEs of 13.3% and 17.8%, respectively. It should be noted that the reported testing MAPE and RMSE are dependent upon the random selection of test points; a new train-test split would result in predictions different from those presented. However, the cross-validation study ensures that the models chosen are as robust as possible to unseen data. Training was also performed using nested cross-validation; however, the results (Table S2) did not show improvement over LOO cross-validation and thus were not considered. We also note that Gaussian Process Regression (GPR), a widely used method to analyze small data sets, did not provide trends consistent with experimental data and was thus not considered in the following (Table S3). To further examine the generalizability of the trained models, they were applied to predict the *E*_norm_(*N*, σ) for an independent data set of polystyrene-grafted silica brush particles (SiO_2_–PS) (final column of Table 3). Polystyrene was chosen for comparison since it is a polymer glass (both polymers are predominantly atactic if synthesized using ATRP) with similar thermomechanical properties to PMMA as indicated by the similar glass transition temperatures of the linear reference polymers (*T*_g_^PS^ = 103 °C; *T*_g_^PMMA^ = 109 °C).^36^ The larger MAPE and RMSE values (Table 3), revealed that models (trained on SiO_2_–PMMA data) captured the principal trend of SiO_2_–PS analogues, albeit at greater error. Random forest (RF) and decision tree regression (DT) were found to provide best transferability, although the difference between both models was blurred by the larger overall error.

Figure 2 compares heatmaps representing the predictions of MLP, KNN, RF, and XGB across the input space for SiO_2_–PMMA systems (the remaining model predictions are shown in the Supporting Information, Figure S1). All models were trained by using the same training data for comparison.

**Figure 2:**
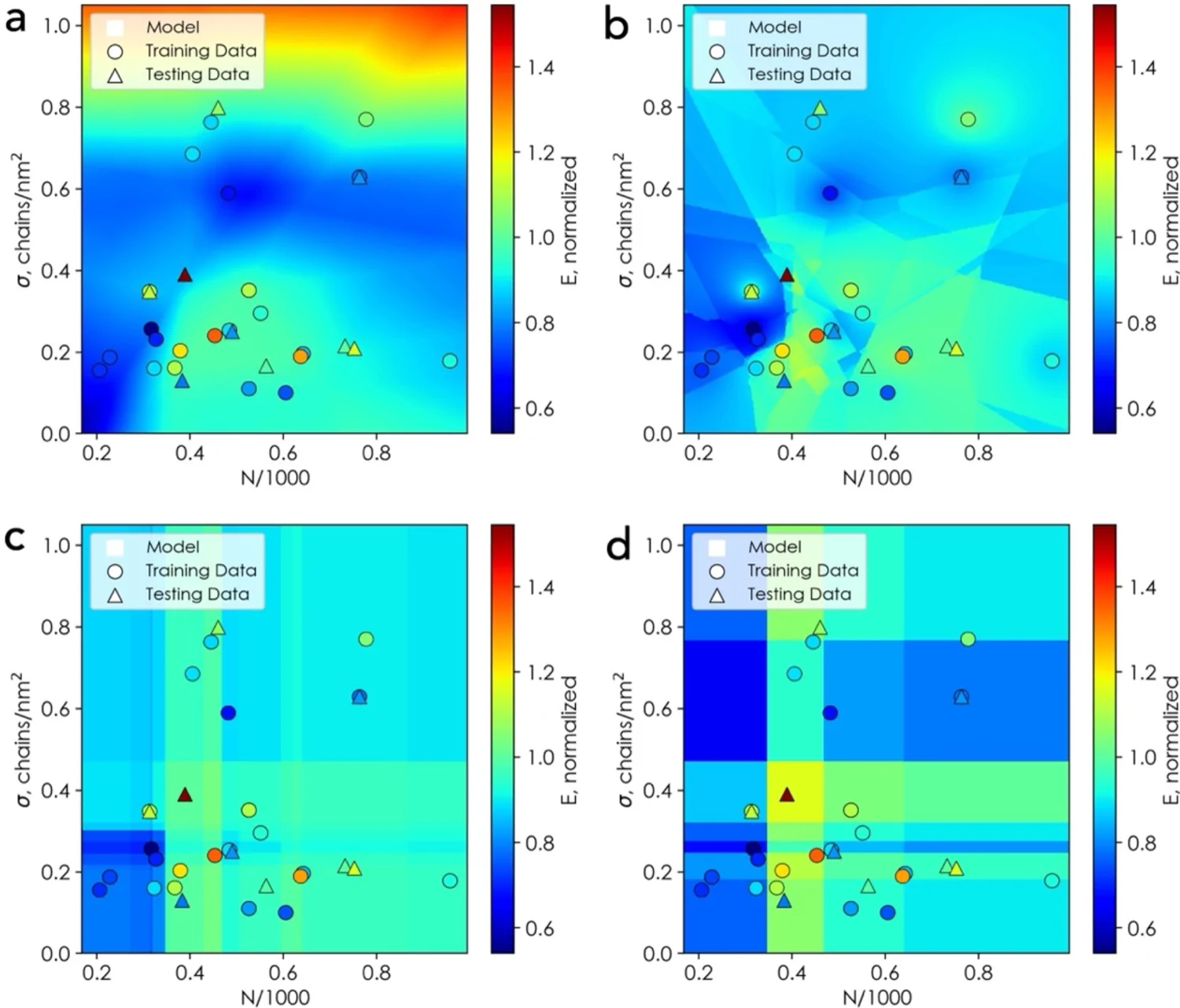
Heatmaps of predicted Young’s modulus *E* for SiO_2_–PMMA with training and testing data points included, over the parameter space σ vs *N* for (a) multilayer perceptron; (b) *k*-nearest neighbor; (c) random forest; and (d) XGBoost models. RF and XGB models predict the most pronounced increase of Young’s modulus with *N* across the intermediate grafting density regime.

In general, the models tended to underfit, rather than overfit. Some of this underfitting is extreme; the benchmark regression techniques including OLS and PR gave simple linear relationships that are nearly uniform across the parameter spaces. Several regression models similarly underfit the data. SVR, for example, predicted *E* as being uniform across the parameter space. The DT model contained only one tree split, to similar effect. In other cases, such as the MLP, which has the capability to represent highly detailed outputs perfectly (sometimes causing undesirable overfitting), overall trends were captured, but intricate variations in the training data were smoothed over.^40^ The underfitting favored by models most likely stems from the validation process: by performing LOO cross-validation, hyperparameters were selected such that models were able to make predictions on unseen validation data with MAPEs as small as possible. The tendency to underfit, therefore, is evidence of the data set’s low signal-to-noise ratio, and validation encourages models to make smooth, simple predictions rather than sharp, complex ones.

Despite the MLP’s ability to capture dynamic changes in the parameter space, the heatmap of its predictions in Figure 2a is decidedly less feature-rich than other regression methods and fails in predicting the rise of modulus with *N* that has also been observed in similar materials using indentation experiments.^35,36,41^ Rather, the model predicted the elastic modulus to be independent of *N* for a wide range of grafting densities. Since it has been shown that cohesive interactions in particle brush assembly structures (for unpolar surfactants) are dominated by van der Waals interactions between ligands, this prediction counters the expectation that Young’s Modulus should trend with increasing number density of polymer segment contacts.^3,33^ A similar conclusion applies to the KNN regression model that did not provide an interpretable trend for the influence of chain length and grafting density on the Young’s Modulus of brush particle films. In contrast, both RF and XGB regression models predicted an increase of the elastic modulus with *N* regardless of grafting density. More importantly, at constant degree of polymerization, both models predicted Young’s Modulus to be largest within the range of intermediate grafting densities, i.e., 0.3–0.5 nm^*–*2^. This is an intriguing prediction since such a trend has indeed been observed in indentation experiments where it was attributed to the reduction of brush interdigitation in the limit of high grafting density and the associated reduction of the number of segment contacts between adjacent brush particles.^36^ Thus, for a given *N* of grafted chains, *E* increased with grafting density in the limit of intermediate grafting density (where brush interdigitation is unhindered), while for high grafting densities (i.e., more dense brush architectures) a reduction *E* with grafting density was observed. The result most consistent with physical intuition and reported experimental trends was provided by XGB (Figure 2d) which also consistently predicted the modulus to increase with the degree of polymerization of grafted chains. Hence, XGB was selected as the best performing model in the group. Figure 3 displays the prediction performance of XGB (trained on PMMA data) in terms of iteration efficacy as well as correlation between training and testing data.

**Figure 3:**
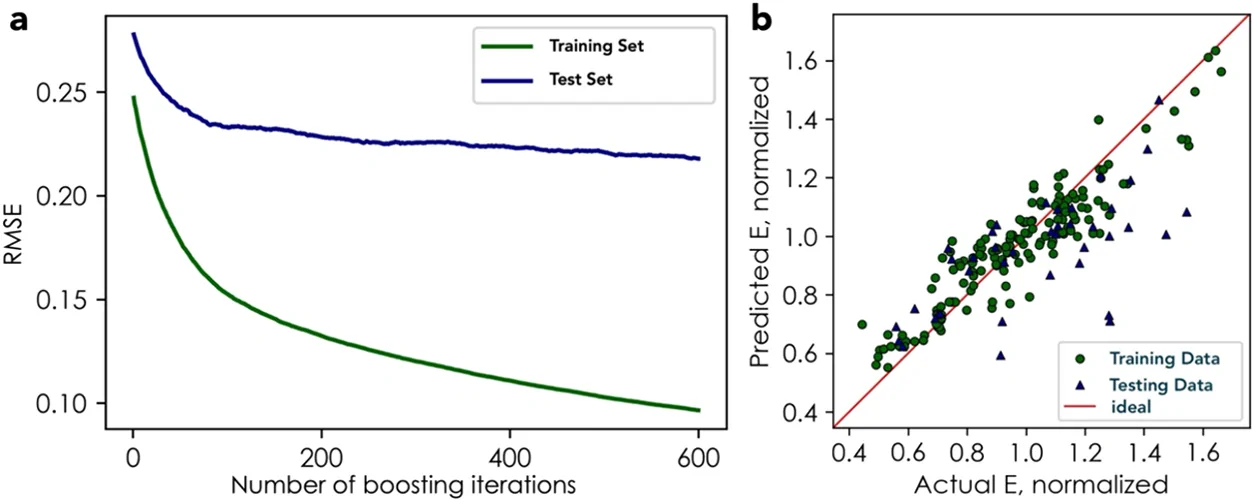
Prediction performance of XGB trained on the PMMA data set (LOO cross-validation). Panel (a) shows the accuracy gain with iterations, and panel (b) shows correlation between predicted and experimental data points.

To evaluate the confidence intervals for predictions, the complete workflow was repeated over 200 independent random 80/20 splits and the variance was corrected to account training set overlap as described by Nadeau and Bengio.^42^ The results revealed standard deviations in the range of 4 to 6 percentage points for every model which was considered satisfactory, given the limited amount of data (Figure S2). It is noted that although XGB provided a physically interpretable prediction of *E*, a significant RMSE and MAPE remained even after 600 iterations (Figure 3a). This is likely attributable to the underlying data set (Table 1) which was limited in both volume and compositional range. It is important to note that such limitations are not easily addressed by chemistry. This is because the accessible resolution and range of material parameters was limited by the kinetics of the polymerization process, which, for a given catalyst system, depended primarily on the choice of monomer. A practical strategy for increasing range and resolution of data is thus the pooling of property data across disjunct polymer systems. We reasoned that a requisite for successful training on pooled data would be the similarity of the respective polymers in terms of microstructure and thermomechanical properties. Hence, the normalized data sets of PMMA and PS were pooled to increase the range of the data set used for training. PMMA and PS were suitable candidates for data pooling since both polymers featured an amorphous structure and similar glass transition temperature—both indicative of a similar physics underlying structure–property relations. Figure 4 shows a heatmap illustrating the *E-N*-σ relation predicted by training the XGB model on the combined data set (Tables 1 and 2) following the same training process as for individual PMMA and PS analogs.

**Figure 4:**
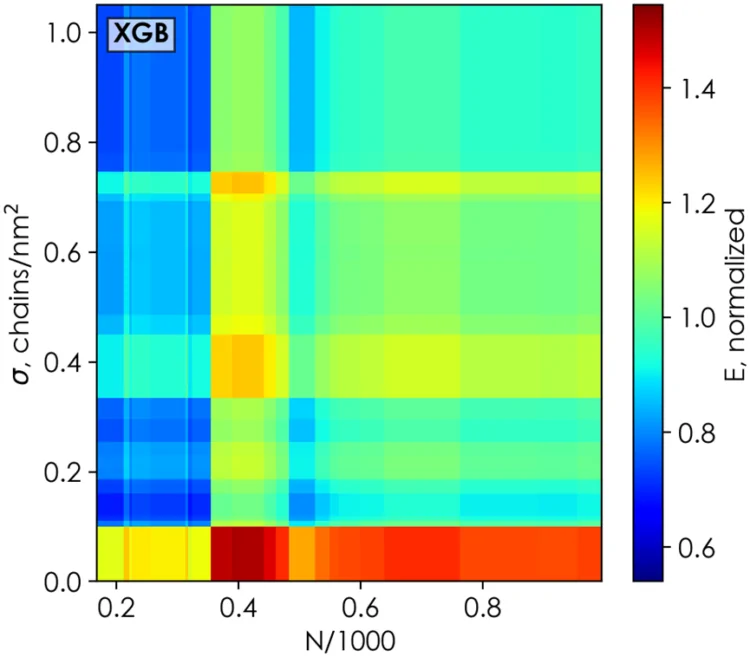
Heatmap of Young’s modulus *E* over the parameter space σ vs *N* predicted using the XGB model after training on the combined data sets of the normalized modulus (Tables 1 and 2). The model predicts the highest modulus in the regime of low grafting densities (σ < 0.1 nm^–2^).

XGB regression using the combined data set suggested a more nuanced dependence of the elastic modulus on the grafting density as compared to the model trained on only SiO_2_–PMMA data. In particular, a region of “high modulus” in the limit of very small grafting density was predicted. Table 4 summarizes the relevant error metrics of the model with respect to PMMA/PS and PS testing data. As for training on the PMMA data set (Table 3), the KNN and XGB regression models were most successful in capturing experimental trends. Particularly notable was the error reduction for PS data prediction, while the accuracy of PMMA data prediction remained similar (albeit somewhat reduced).

**Table 4:** MAPE and RMSE of Each Model on Training (PMMA+PS), Testing (PMMA+PS), and PS Data^a^

	train (PMMA/PS)		test (PMMA/PS)		test (PS)	
model	MAPE	RMSE	MAPE	RMSE	MAPE	RMSE
DT	21.88%	0.226	24.08%	0.269	23.37%	0.236
KNN	**10.21%**	**0.168**	**11.19%**	0.191	**5.33%**	**0.127**
MLP	13.51%	0.178	15.11%	0.192	11.12%	0.170
OLS	21.99%	0.237	24.43%	0.271	24.05%	0.269
PR	22.25%	0.239	24.49%	0.269	24.02%	0.269
RF	17.61%	0.191	19.99%	0.240	18.08%	0.189
SVR	18.63%	0.216	20.50%	0.241	17.82%	0.212
XGB	16.13%	0.177	15.69%	**0.187**	15.78%	0.172

a Abbreviations: DT, decision tree; KNN, *k*-nearest neighbor; MLP, multilayer perception; OLS, ordinary least-squares; PR, polynomial regression; RF, random forest; SVR, support vector regression; and XGB, extreme gradient boosting.

To illustrate the significance of the “high modulus” region in the limit of small grafting density that was predicted by the XGB training on pooled data (Figure 4), the model predictions were compared against results reported by Lee et al., who measured the Young’s modulus of SiO_2_–PMMA brush particle solids (using nanoindentation) across a wide *N*-σ range.^36^ This data set is notably distinct from the one presented here in that a different chemistry was used to realize brush particles with ultralow (sparse) grafting density. Figure 5 compares the *E-N* relationships predicted by XGB and PR with the experimental data for the dense (σ ∼ 0.7 nm^–2^, Figure 5a), intermediate (σ ∼ 0.16 nm^–2^, Figure 5b), and sparse (σ ∼ 0.04 nm^–2^, Figure 5c) grafting regime. In the figure, “XGB” refers to training based on only PMMA data (Table 1), while “XGB-2” refers to training using the combined SiO_2_–PMMA/PS data set (Tables 1 and 2). Absolute values were predicted by multiplication of normalized data (Figure 4) with the elastic modulus of a linear PMMA reference polymer (*E* = 3.8 GPa) as reported by Lee et al.^36^

**Figure 5:**
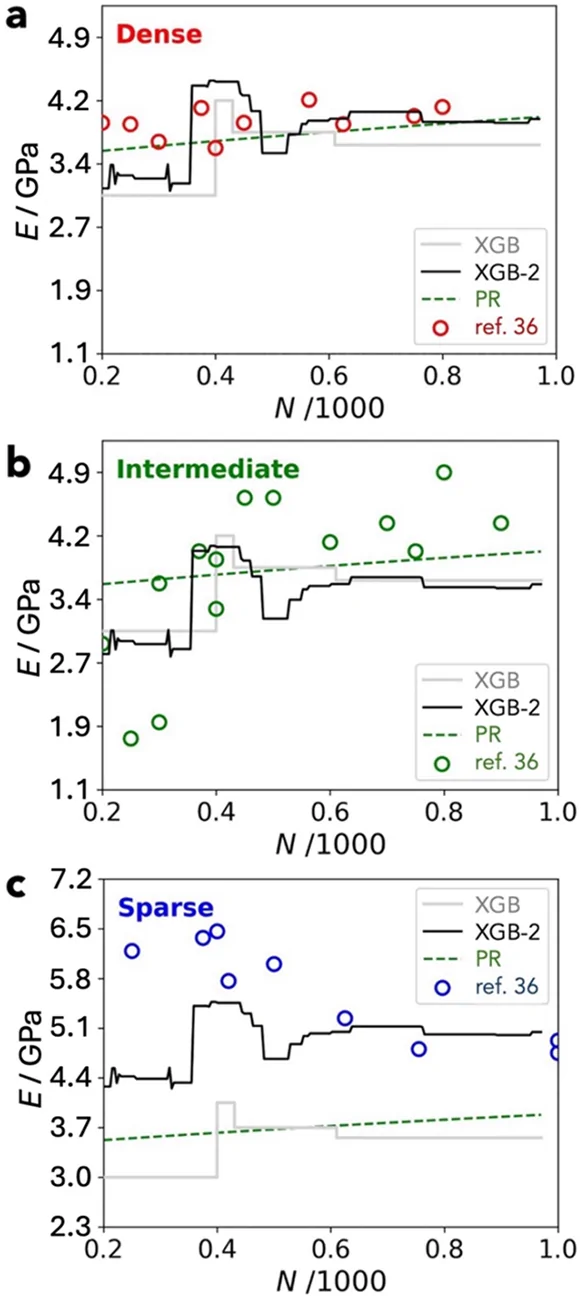
Comparison of the predicted (lines) and measured (circles) dependence of normalized modulus on the degree of polymerization *N* for distinct grafting density regimes: dense (σ *∼* 0.7 nm^–2^, panel a), intermediate (σ *∼* 0.16 nm^–2^, panel b), and sparse (σ *∼* 0.04 nm^–2^, panel c) for SiO_2_PMMA. Data reproduced from ref 36. Copyright [2020] American Chemical Society. Data sets used for training XGB model are SiO_2_–PMMA (“XGB”, gray line) and PMMA/PS (“XGB-2”, black line). The combination of data sets (SiO_2_–PMMA/SiO_2_–PS) significantly improves the XGB prediction in the sparse grafting regime.

The figure reveals that both models (XGB, XGB-2, PR), irrespective of whether training was performed on the PMMA or PMMA/PS data set, qualitatively captured the trend of experimental data in the dense and intermediate grafting regime. However, PR (and similar OLS, not shown here) predicted the normalized modulus to continuously increase with *N* across all grafting regimes which was not consistent with the experimental trend which displayed a more “peaked” behavior that was better captured by the XGB models. Interestingly, whereas both PR and XGB (when trained on only the PMMA data) failed to capture the observed increase of Young’s modulus in the sparse grafting regime, the XGB model trained on the combined data set correctly predicted the uptick of *E* in the sparse grafting regime (“XGB-2” in Figure 5c). The uptick of *E* was indeed observed in experiments and rationalized as the consequence of a microstructure transition toward string-like particle arrays; the increase of particle-to-particle contacts was argued to be responsible for increase the material’s resistance to deformation.^16,36^ The ability of the XGB model to capture the experimental trend, in spite of the change in “the physics” underlying the material’s elastic response, is intriguing and illustrates the potential benefits associated with the use of more complex regressor models, such as XGB, for material property prediction. The result also demonstrates that the pooling of data across disjunct polymer systems (with distinct polymerization kinetics) can provide a path to not only increase the size of data sets but also to broaden the relevant parameter range to enable better forecasting.

## Conclusions

Regression provides a versatile empirical means to predict structure–property relations in complex materials with properties that are governed by multiple parameters with unknown interrelationships. However, the application of regression models is often complicated by experimental data being sparse, limited to narrow ranges, or noisy. Advances in data science and machine learning have expanded the toolset for empirical regression of experimental data to include estimation methods that do not make assumptions about functional relationships between experimental outcomes. For the example of brush particle solids composed of PMMA- and PS-grafted silica nanoparticles with varying grafting density and degree of polymerization of tethered chains, regression methods such as *k*-nearest neighbors or XGBoost outperform established methods such as linear or polynomial regression. In addition to better predicting the trends of experimental data, XGBoost and *k*-nearest neighbors also captured expected trends of parameter variations and featured better transferability to other material compositions such as PS-grafted brush particle analogues. Pooling of data across multiple material systems such as PMMA- and PS-grafted silica provided a means to expand the range of testing and training data and enabled the prediction of property changes in parameter spaces even outside the range of the primary data set. It is expected that the success of such data pooling is contingent on materials sharing characteristics, such as solid-state structure or thermomechanical properties. To understand the role of material characteristics on the quality of predictions based on multimaterial data sets provides an opportunity for future research. We note that the results of this study should not be understood as proof of the better performance of machine learning methods to property prediction, nor that generalizability to other structure–property relationships can be assumed. This is because machine learning methods feature an internal bias that sensitively depends on the density and range of data used to train the models. Opportunities for future research thus include the systematic variation of training and testing data sets (either by application of synthetic conditions that allow broader compositional ranges or the pooling of data related to distinct polymer compositions), the development of synthetic protocols enabling the testing of wider compositional ranges, and the reduction of the dependence on large experimental data sets without reducing the accuracy of predictions, e.g., by integration of molecular dynamics (MD) simulation of data.

## Supplementary Material



## Notes

Model hyperparameters; additional model prediction visualizations; and distribution of the test errors over 200 random splits (PDF)
