# Supplementary material for: Machine Learning Regression-Enhanced Property Prediction of Brush Particle Solids
Source: ACS Appl Polym Mater. 2026 Oct 13;8(20):18740–9. doi: 10.1021/acsapm.6c03454 (PMC13711683; doi:10.1021/acsapm.6c03454)
Supplement: Supplementary file 1 [file ap6c03454_si_001.pdf]

Supporting Information

Machine Learning Regression Enhanced Property Prediction of Brush Particle Solids

Kevin Ferguson[†], Ayesha Abdullah[‡], Yuqi Zhao[‡], Yunhao Xu[‡], Michael R. Bockstaller[*, ‡], Levent Burak Kara[†], Lawrence Drummy[¶], Eric Harper[¶], Andrew Gillman[¶]

† *Department of Mechanical Engineering, Carnegie Mellon University, 5000 Forbes Ave, Pittsburgh, PA 15213*

‡ *Department of Materials Science and Engineering, Carnegie Mellon University, 5000 Forbes Ave, Pittsburgh, PA 15213*

¶ *Materials & Manufacturing Directorate, Air Force Research Laboratory, 2977 Hobson Way, Wright-Patterson AFB, OH 45433*

*) Corresponding author E-mail: bockstaller@cmu.edu

Phone: +1 412 268 2709 Fax: +1 412 268 7247

1. Definitions

Mean Average Percent Error (MAPE): measures the accuracy of a forecasting method as a percentage. It represents the average absolute percent error between the predicted values and the actual values.

$$MAPE = \frac{100\%}{n} \sum_{i=1}^{n} \left| \frac{y_i - \hat{y}_i}{y_i} \right|$$

where n is the number of observations, y_i is the experimental value, and $\hat{y}_i$ is the predicted value.

Root Mean Squared Error (RMSE): is the square root of the average of squared differences between prediction and actual observation. It measures the standard deviation of the residuals (prediction errors).

$$RMSE = \sqrt{\frac{\sum_{i=1}^{n} (y_i - \hat{y}_i)^2}{n}}$$

Where n is the number of observations, y_i is the experimental value, and $\hat{y}_i$ is the predicted value.

2. Leave-One-Out Cross Validation

Table S2. Options considered and selected for each hyperparameter during leave-one-out cross-validation.

Decision Tree (DT)			
Parameter	Argument Name	Options	Selected
Maximum Depth	'max_depth'	1, 2, 3, 4, 5, 6	1
K-Nearest Neighbors (KNN)			
Parameter	Argument Name	Options	Selected
Number of Neighbors	'n_neighbors'	1, 2, 3, 4, 5	5
Weight Type	'weights'	'uniform', 'distance'	'distance'
Multilayer Perceptron (MLP)			
Parameter	Argument Name	Options	Selected
Hidden Layer Sizes	'hidden_layer_sizes'	[32], [32,32], [32,32,32], [64], [64,64], [64,64,64]	[64, 64]
Activation	'activation'	'tanh', 'relu'	'relu'
Maximum Iterations	'max_iter'	-	10000
Initial Learning Rate	'learning_rate_init'	-	1.00E-09
Tolerance	'tol'	-	0.00005
Ordinary Least Squares (OLS)			
Parameter	Argument Name	Options	Selected
-	-	-	-
Polynomial Regression (PR)			
Parameter	Argument Name	Options	Selected
Polynomial Degree	'degree'	2, 3, 4, 5	2
Ridge Regression alpha	'alpha'	0, 0.1, 0.25, 0.5, 1.0, 2.0, 4.0	4
Random Forest (RF)			
Parameter	Argument Name	Options	Selected
Number of Estimators	'n_estimators'	8, 16, 32, 64	16
Maximum Tree Depth	'max_depth'	2, 4, 6, 8, 10, 12	2
Support Vector Machine (SVM)			
Parameter	Argument Name	Options	Selected
Inv. Reg. Strength	'C'	1e-2, 1e-1, 1, 1e1, 1e2, 1e3, 1e4, 1e5	1.00E-02
Kernel	'kernel'	-	'rbf'
Tolerance	'tol'	-	1.00E-09
XGBoost (XGB)			
Parameter	Argument Name	Options	Selected
Learning rate, eta	'eta'	0.001, 0.01, 0.1, 0.5, 0.75, 1.0	1

Maximum Depth	'max_depth'	1, 2, 4, 8, 16	1
Number of Estimators	'n_estimators'	16, 32, 48, 64	16

3. Nested Cross-Validation

To evaluate the effect of cross-validation method, 'nested cross-validation' was performed. The process involves the initial splitting of data into outer train/test folds. The outer test fold is kept unseen until final evaluation. Inside each outer-train split, cross-validation is run to choose the optimum hyperparameters. Subsequently, a model is trained with the best inner-loop hyperparameters on the full outer-train data, and then evaluated on the outer-test fold. The process is repeated and results are averaged. Table S2 shows the results of nested cross-validation for the different regression models.

Table S2. Nested Cross Validation Performance.

Model	Train		Test		PS	
	MAPE	RMSE	MAPE	RMSE	MAPE	RMSE
DT	16.16%	0.179948	18.30%	0.195581	16.52%	0.182168
GPR	3.16%	0.039855	19.55%	0.238187	7.19%	0.133552
KNN	4.72%	0.066703	19.18%	0.237182	7.22%	0.140526
MLP	18.23%	0.203315	21.96%	0.243861	19.17%	0.226288
OLS	21.26%	0.236518	22.76%	0.250065	24.19%	0.273632
PR	21.21%	0.234659	22.72%	0.248029	23.89%	0.269854
RF	11.41%	0.12523	18.05%	0.199588	12.74%	0.141957
SVM	18.76%	0.218787	21.25%	0.238231	20.80%	0.249657
XGB	15.26%	0.167189	21.18%	0.226373	17.64%	0.198185

4. Gaussian Process Regression (GPR)

Table S3. Performance Table for Gaussian Process Regression (LOOCV).

Model	Train		Test		PS	
	MAPE	RMSE	MAPE	RMSE	MAPE	RMSE
GPR	17.07%	0.207329	16.37%	0.20903	9.30%	0.125525

5. Heatmaps of predicted Young's modu

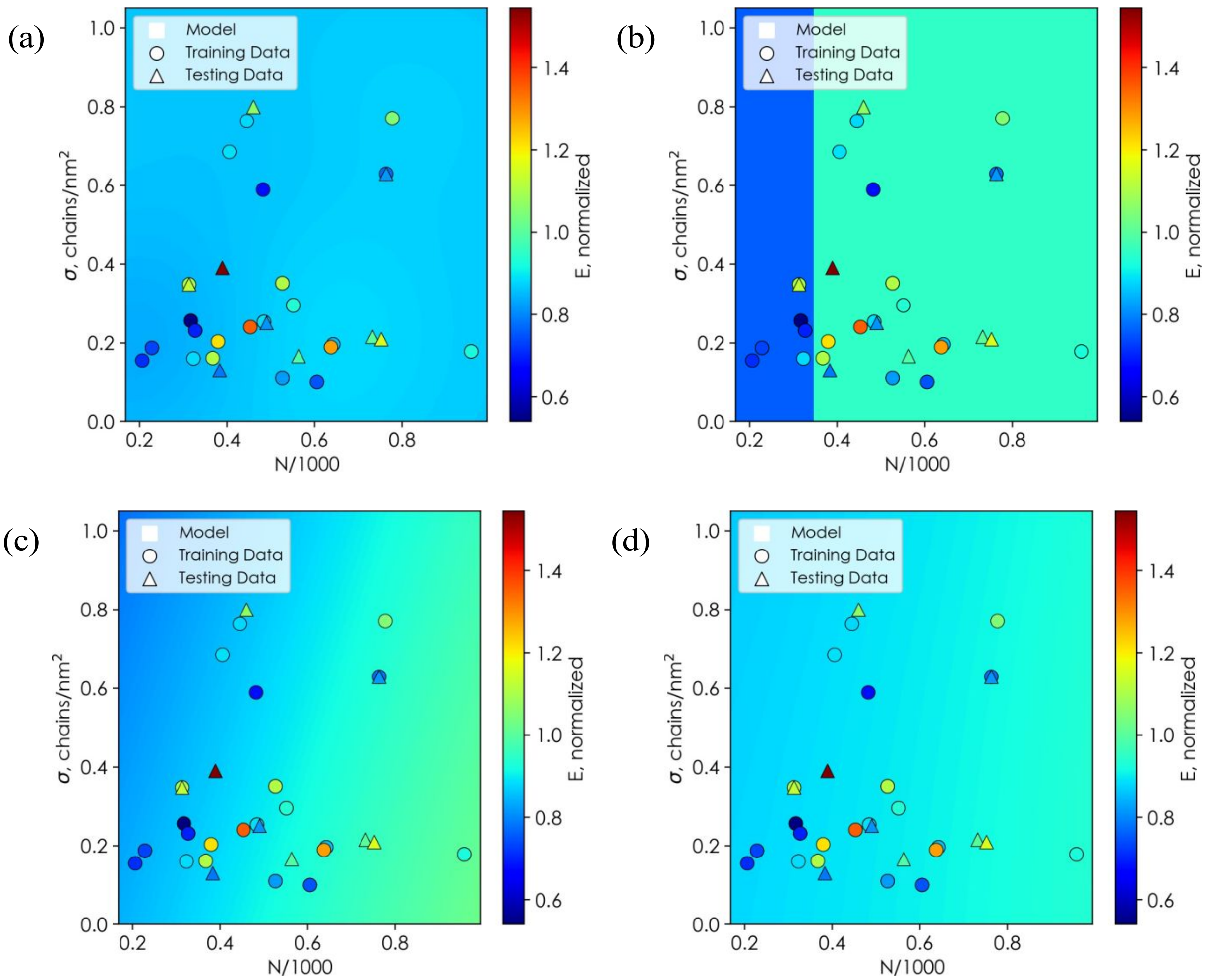

Figure S1. Heatmaps of predicted Young's modulus E with training and testing data points included, over the parameter space σ vs N for (a) support vector machine; (b) decision tree; (c) ordinary least-squares; and (d) polynomial regression.

6. Confidence Intervals of Models

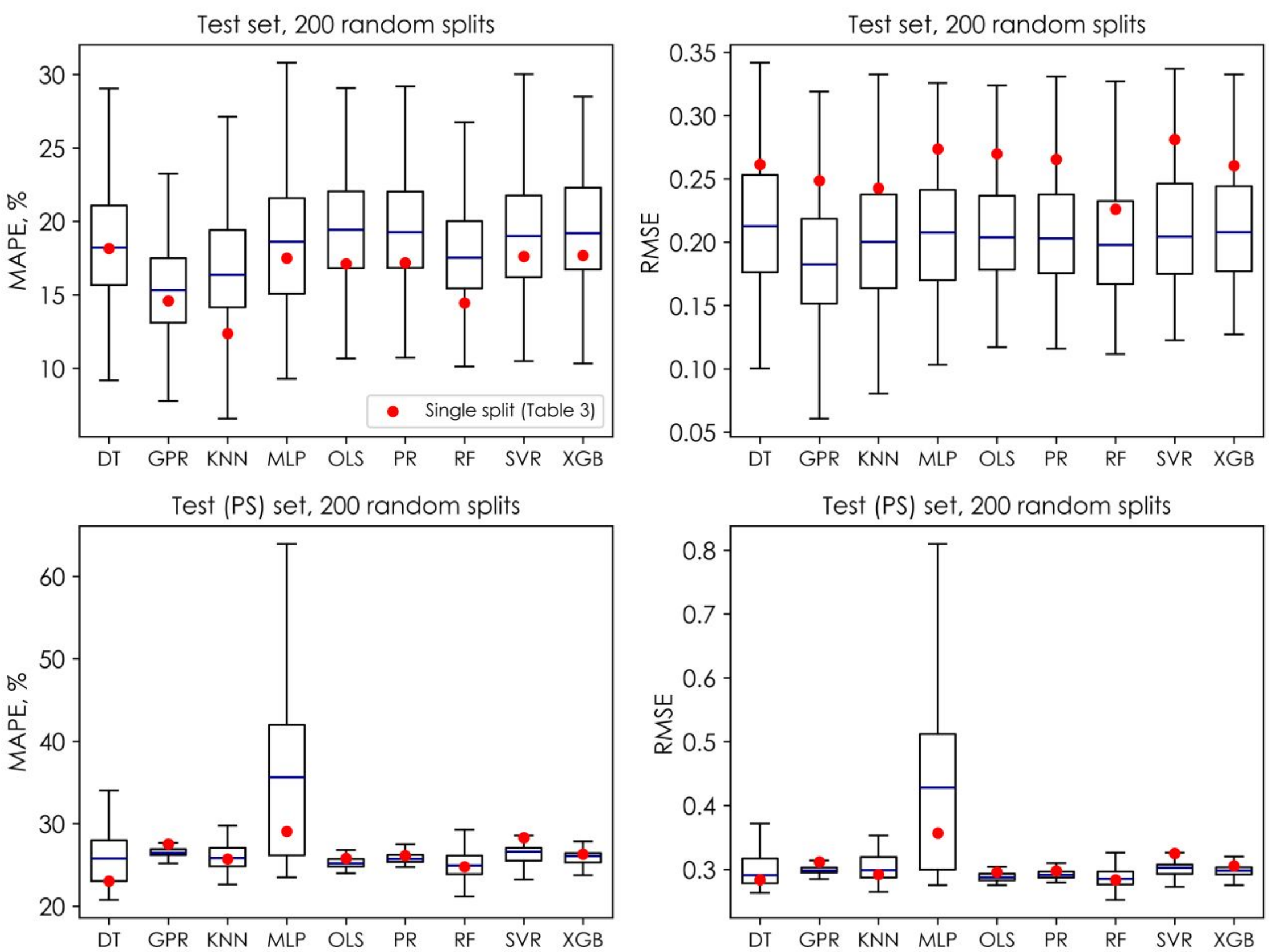

Figure S2. Distribution of the test errors over 200 random splits. Red points mark the single split used in Table 3.
